# Supplementary material for: Clinical relevance of low-density Plasmodium falciparum parasitemia in untreated febrile children: A cohort study
Source: PLoS Med. 2020 Sep 21;17(9):e1003318. doi: 10.1371/journal.pmed.1003318 (PMC7505590; doi:10.1371/journal.pmed.1003318)
Supplement: S1 Table — HD, high-density Pf infection; LD, low-density Pf infection; ND, no detectable Pf parasitemia; sd-mRDT, standard malaria RDT; us-mRDT, ultrasensitive-mRDT (Alere); us-qPCR, ultrasensitive quantitative PCR. (DOCX) [file pmed.1003318.s002.docx]

***Table S1.* Results of standard mRDT and ultrasensitive-mRDT stratified by ultrasensitive-qPCR*.***

*sd-mRDT: Standard malaria RDT; us-mRDT: Ultrasensitive-mRDT (Alere); us-qPCR: ultrasensitive quantitative PCR; HD: High-density Pf infection, LD: Low density Pf infection, ND: No detectable Pf parasitaemia.*

|  |  | **us-qPCR negative** *(gold standard)* | | |  |  |  | **us-qPCR positive** *(gold standard)* | | |  |
| --- | --- | --- | --- | --- | --- | --- | --- | --- | --- | --- | --- |
|  |  | **us-mRDT** | | ***subtotal*** |  |  |  | **us-mRDT** | | ***subtotal*** |  |
|  |  | **-** | **+** |  |  |  |  | **-** | **+** |  | **TOTAL** |
| **sd-mRDT** | **-** | 2519 | 7 | *2526* |  | **sd-mRDT** | **-** | 73 | 3 | *76* **LD:** us-qPCR-positive cases missed by sd-mRDT | **2602** |
|  | **+** | 0 | 1 | *1* |  |  | **+** | 0 | 198 | *198* **HD:** sd-mRDT-positive cases confirmed by us-qPCR | **199** |
|  | | *2519* | *8* | ***2527* ND:** Total us-qPCR-negative samples |  |  |  | *73* | *201* | ***274*** | **2801** |
