# Supplementary material for: Clinical relevance of low-density Plasmodium falciparum parasitemia in untreated febrile children: A cohort study
Source: PLoS Med. 2020 Sep 21;17(9):e1003318. doi: 10.1371/journal.pmed.1003318 (PMC7505590; doi:10.1371/journal.pmed.1003318)
Supplement: S2 Table — Adapted from Keitel et al. [13]. (DOCX) [file pmed.1003318.s003.docx]

**Table S2. Definition of clinical failure (primary outcome measure).** Adapted from Keitel et al. [[13](#_ENREF_13)]

| \| **Clinical failure** \| **At any time after initial assessment** \| Persistence of new onset of “severe illness” ^1^   - Coma - >2 convulsions within 24h - Inability to drink or breastfeed - Tachypnoea ^2^ - Tachycardia ^3^ - Hypoxemia ^4^   Clinical pneumonia ^5^  Severe dehydration ^8^ \| \| \| --- \| --- \| --- \| --- \| \| **At day 7** \| Fever or temperature > 38°C  Diarrhoea  Serious skin infection ^6^  A new significant symptom or sign related to the acute episode but not present at day 0 \| \| \| **Anaemia** \| **Moderate-to-severe** \| Haemoglobin >9 g/dl \| \| \| **Severe** \| Haemoglobin >6 g/dl \| **^1^ Severe illness**  any of the conditions highlighted in orange \| \| **Severe malnutrition** \| Weight-for-Age Z-score < -3 and/or MUAC <11.5 cm. \| \| \| **^8^ Severe dehydration** \| Requiring facility-based treatment   - Not tolerating oral liquids - Tachycardia \| \| \| **Severe pneumonia** \| Clinical pneumonia ^5^ AND (Severe respiratory distress ^7^ OR severe tachypnoea ^2^ OR Hypoxemia ^4^) \| \| \| **Suspicion of meningitis** \| Severe lethargy OR neck stiffness OR convulsions \| \| \| **^2^ Tachypnoea** \| Respiration rate > 97th percentile for age and temperature \| \| \| \| **^3^ Tachycardia** \| Heart rate > 90th percentile for age and temperature \| \| \| \| **^4^ Hypoxemia** \| Oxygen saturation (SaO2 < 90%) \| \| \| \| **^5^ Clinical pneumonia** \| Either (cough AND tachypnoea ^2^) OR (cough AND chest indrawing) \| \| \| \| **^6^ Serious skin infection** \| Requiring antibiotic based treatment \| \| \| \| **^7^ Severe respiratory distress** \| Speaks only single words OR grunts OR speaks short phrases only OR short cries AND chest indrawing \| \| \| |
| --- | --- | --- | --- | --- | --- | --- | --- | --- | --- | --- | --- | --- | --- | --- | --- | --- | --- | --- | --- | --- | --- | --- | --- | --- | --- | --- | --- | --- | --- | --- | --- | --- | --- | --- | --- | --- | --- | --- | --- | --- | --- | --- | --- | --- | --- | --- | --- | --- | --- | --- |
