# Supplementary material for: Clinical relevance of low-density Plasmodium falciparum parasitemia in untreated febrile children: A cohort study
Source: PLoS Med. 2020 Sep 21;17(9):e1003318. doi: 10.1371/journal.pmed.1003318 (PMC7505590; doi:10.1371/journal.pmed.1003318)
Supplement: Attachment — Submitted filename: hartley.pdf [file pmed.1003318.s004.pdf]

This paper describes a secondary analysis of data on parasitaemia in febrile children in Tanzania.

I was asked for a statistical report and I interpret that to include all aspects of the design and conduct of the study.

Points of detail

Title I debated for some while whether retrospective is correct here. The data-set was collected, as I understand it, prospectively and this is a secondary analysis. I think calling it retrospective is doing it a disfavour and I would rephrase it or just drop retrospective.

Page 4 Perhaps mention the trial already here rather than wait to the methods?

Page 6 I think I might make explicit here what the arms were in the trial. I know we can find out from Figure 1. This does raise the important question of whether in the re–analysis of data from a trial one should always include the arm as a confounder. I think it might be safer.

Page 7 'who were rather called at day 7' is probably more idiomatic in some other language (?German). Perhaps 'were followed by telephone at day 7' assuming I understand it correctly.

Page 8 If you have the data and want risk ratios then why not use a log binomial model rather than an approximate method?

Page 9 I think the actual amount of missing data is strictly speaking part of the results. The authors did not plan to have 1374 children without anaemia. I do not think the authors can justify the statement that anaemia is MCAR. When I look at Table 4A and B I see that D status predicts anaemia. I think a re–think is needed here and possibly extended analysis.

Page 15 In general, and especially here, it is better to use a comma or the word 'to' in order to separate the limits of a confidence interval as initially I mis–read -2.6-2.9 as two negative numbers. This is obviously less critical for the risks which are guaranteed to be non–negative although I think it would be best to be consistent.

Page 15 I am not sure what a directly proportional linear correlation is but when I look at Figure 3B I do not see a straight line. I assume the reversal at the very low ages reflects low numbers of very young

children which might be confirmed for us. Perhaps even a violin plot of child ages?

Page 15 Polynomials have some properties which may be undesirable as they depend on non–local behaviour (Magee, 1998). This may not matter too much if the intention here is a purely descriptive one but splines might be better even though they are not problem–free either.

Page 19, Figure 3 For plots B and D box–plots or violin plots as in Figure 4 would be better. Given the small number of children in the right hand groups I might even consider dot–plots.

Page 20, Figure 4 Sub–figure B looks very strange as though CRP is only measured to the nearest multiple of 20. There could be some sort of digit preference explanation I suppose. What is going on here?

Page 21, Table 4 Even though the different N for sickle cell is explained earlier the reader may have forgotten reading it (I had) so I might repeat it in the caption.

Page 23 Figure 5C seems to be missing.

Page 25, Table 5 I am not sure whether the differences in deaths are particularly important but it would be possible to present a risk difference with its confidence interval or a confidence interval for the individual proportions.

Points of more substance

Model selection

On page 8 the authors tell us that three variables (age, seasonality, and malnutrition) are considered on theoretical grounds to be confounders. Although it is not explicit it seems, for example from Table 3, that some form of variable selection was used here. Selecting a subset of variables in a way driven by the data leads to a model which is unlikely to replicate (Babyak, 2004). Screening variables to enter the model has been criticised too (Sun et al., 1996). Why not fit the adjusted models using all the specified confounders? Surely in this sort of study we want to say that the effect persisted in spite of our best efforts to adjust it away rather than being able to say that it is not necessary to measure some of the confounders. If the authors feel it is necessary then state why and also state what method was employed to select

the variables: forward selection, backward elimination, best ossible subset, ...

No evidence of effect versus evidence of no effect

I felt that in a number of places the authors do not place enough stress on the fact that with 76 children in the LD group some of the confidence intervals are quite wide and we need to be more cautious about sliding from saying there is no evidence of difference to saying there is no difference.

Summary

Points for clarification and expansion and some suggested changes to analysis.

Michael Dewey

References

M A Babyak. What you see may not be what you get: a brief, nontechnical introduction to overfitting in regression–type models. *Psychosomatic Medicine*, 66:411–421, 2004.

L Magee. Nonlocal behavior in polynomial regressions. *American Statistician*, 52:20–22, 1998.

G-W Sun, T L Shook, and G L Kay. Inappropriate use of bivariable analysis to screen risk factors for use in multivariable analysis. *Journal of Clinical Epidemiology*, 49:907–916, 1996.
