# Supplementary material for: Clinical relevance of low-density Plasmodium falciparum parasitemia in untreated febrile children: A cohort study
Source: PLoS Med. 2020 Sep 21;17(9):e1003318. doi: 10.1371/journal.pmed.1003318 (PMC7505590; doi:10.1371/journal.pmed.1003318)
Supplement: Attachment — Submitted filename: UltrasensitiveMalariaDiagnostics(Hartley_09June20)_Reviewer.pdf [file pmed.1003318.s005.pdf]

unisanté EPFL

09 June 2020

RE: Response to Reviewers for the manuscript entitled: *Clinical relevance of low-density Plasmodium falciparum parasitaemia in untreated febrile children: a prospective cohort analysis.*

Dear PLoS Medicine team,
Dear Reviewers

We sincerely thank the reviewers for this thorough, constructive and positive evaluation of our manuscript.

We are happy to report that we have been able to address all issues raised and feel that the revised version has been greatly improved by the process.

Please find a point-by-point response to the comments below.

Reviewer 1
We thank the reviewer for his valuable insight. Particularly in identifying the accidental misclassification of the study as retrospective, when indeed we had declared the aims prospectively! (IRB protocol provided above)
Extremely useful comments that will also help guide my future analyses. Much appreciated.

**Comment**	**Response**
Title I debated for some while whether retrospective is correct here. The data-set was collected, as I understand it, prospectively and this is a secondary analysis. I think calling it retrospective is doing it a disfavour and I would rephrase it or just drop retrospective.	Indeed!<br>This is, in fact, a prospective analysis. Some confusion arose as the ultrasensitive malaria tests were performed after the outcome was recorded. However, these tests were performed on blood samples originating from day 0 of the follow-up period and the aim of investigating aetiologies of non-malarial fever was described in the original IRB protocol.<br>Thus, we investigate the effect of an exposure (low-density malaria parasitaemia) during longitudinal follow-up.<br>***TITLE:*** *Clinical relevance of low-density Plasmodium falciparum parasitaemia in untreated febrile children: a prospective cohort analysis.*
Page 4 Perhaps mention the trial already here rather than wait to the methods?	Agreed. Added as suggested.
Page 6 I think I might make explicit here what the arms were in the trial. I know we can find out from Figure 1. This does raise the important question of whether in the re–analysis of data from a trial one should always include the arm as a confounder. I think it might be safer.	Agreed. This was actually tested and should have been reported. The arm posed no effect on the outcomes investigated.<br><br>As the analyses tables are already critiqued elsewhere for their complexity, we will rather specify this in a sentence in the methodology section.<br>***MATERIALS AND METHODS (Design, Patient population and context)***<br>*The arm of the trial did not affect any outcomes investigated.*
Page 7 'who were rather called at day 7' is probably more idiomatic in some other language (?German). Perhaps 'were followed by telephone at day 7' assuming I understand it correctly.	Agreed and corrected.

Page 9 I think the actual amount of missing data is strictly speaking part of the results. The authors did not plan to have 1374 children without anaemia. I do not think the authors can justify the statement that anaemia is MCAR. When I look at Table 4A and B I see that D status predicts anaemia. I think a re–think is needed here and possibly extended analysis.	Agreed.<br>The text of this section did not appreciate the important difference between MCAR and MAR<br>• MCAR (Missing completely at random): no pattern in the missing data on ANY variables.<br>• MAR (missing at random): missing randomly across the cohort groups.<br><br>The features are not MCAR, as indeed it is collected according to triage criteria which naturally biases it according to clinical presentation groups.<br>However, the clinical presentation groups were equally represented in each sub-cohort and thus missing at random (MAR) with respect to the outcome categories.<br>For the example highlighted by the reviewer (anaemia) Missingness of this feature between the cohort groups are<br>• HD and LD: 47%vs 40% respectively, p=0.2<br>• ND and LD: 49%vs 40% respectively, p=0.1<br><br>Missingness of other features are negligible.<br><br>The section is included in the methods and not as results as these analyses have already been performed as part of data pre-processing in this study as well as in previous study on the prospective analysis (Keitel et al PLoSMed, 2018).<br><br>We have now added these considerations to the text.
	MATERIALS AND METHODS (Statistical analyses, missing values)<br>*Missing values were missing at random according to the three subgroups (LD, HD, ND) and were thus excluded from analyses.*<br>*We present the missingness testing for the measurement of haemoglobin as it was particularly affected with only 51% of the values collected. Missingness was non-significant between both HD vs LD (47% vs 40% respectively, p=0.2) and ND vs LD (49% vs 40% respectively, p=0.1)*
Page 15 In general, and especially here, it is better to use a comma or the word 'to' in order to separate the limits of a confidence interval as initially I mis–read -2.6-2.9 as two negative numbers. This is obviously less critical for the risks which are guaranteed to be non–negative although I think it would be best to be consistent.	Agreed.<br>All "-" have been to changed to semi-colons with a space for reporting CI95%.<br><br>Semi-colons are now only used for CI95% intervals and commas are used to separate other statistical parameters.<br><br>The manuscript and all tables have been checked for consistency on this issue.
Page 15 I am not sure what a directly proportional linear correlation is but when I look at Figure 3B I do not see a straight line. I assume the reversal at the very low ages reflects low numbers of very young children which might be confirmed for us. Perhaps even a violin plot of child ages?	The wording "directly proportional linear" is now removed.
Page 15 Polynomials have some properties which may be undesirable as they depend on non–local behaviour (Magee, 1998). This may not matter too much if the intention here is a purely descriptive one but splines might be better even though they are not problem–free either.	We thank the reviewer for the interesting reference and agree with their conclusion.<br><br>Indeed the first figures of this paper are purely descriptive statistics which aim to allow the reader to get a feel for the scope and range of data collected.
Page 19, Figure 3 For plots B and D box–plots or violin plots as in Figure 4 would be better. Given the small number of children in the right hand groups I might even consider dot–plots.	Agreed.<br>The figures are now replaced with dot-plots and are much easier to read.

	This also allowed us to correct the cohort numbers which became unlinked to the numbers in the table, and rather indicated the value in an adjacent cell.
	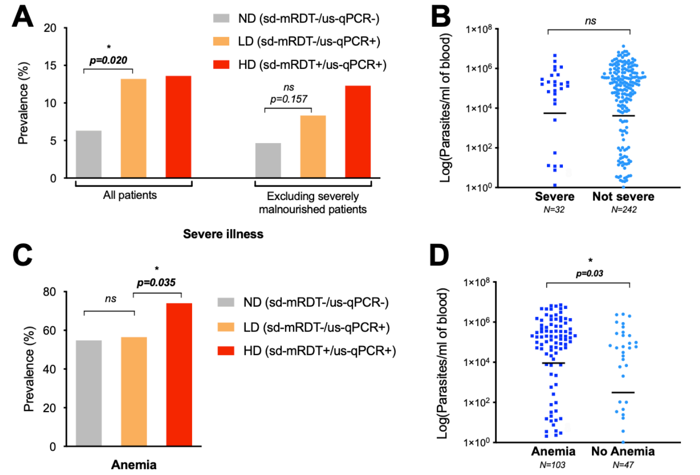
Page 20, Figure 4 Sub–figure B looks very strange as though CRP is only measured to the nearest multiple of 20. There could be some sort of digit preference explanation I suppose. What is going on here?	Indeed. The CRP rapid diagnostic tests are measured in pre-binned units<br>We have taken the lower bound as a marker<br>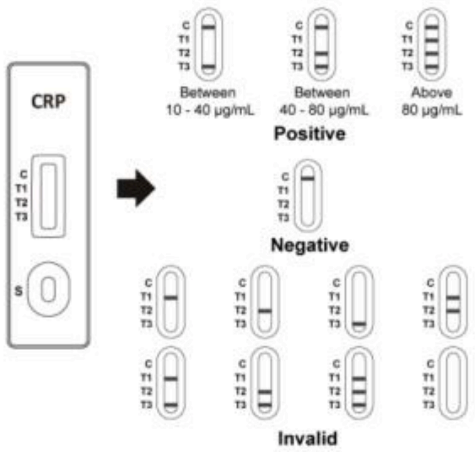<br>This is explained in the legend.<br>*CRP was measured using a categorical quantitation test. Four categories exist: <10mg/L, 10-40mg/L, 40-80mg/L and >80mg/L. The lower limit of this range is represented in the plot.*
Page 21, Table 4 Even though the different N for sickle cell is explained earlier the reader may have forgotten reading it (I had) so I might repeat it in the caption.	This is actually already in the caption.
Page 23 Figure 5C seems to be missing	Thank-you for spotting this. It was a typo. The reference is now removed.
Model selection On page 8 the authors tell us that three variables (age, seasonality, and malnutrition) are considered on theoretical grounds to be confounders. Although it is not explicit it seems, for example from Table 3, that some form of variable selection was used here. Selecting a subset of variables in a way driven by the data leads to a model which is unlikely to replicate (Babyak, 2004). Screening variables to enter the model has been criticised too (Sun et al., 1996). Why not fit the adjusted models using all the specified confounders? Surely in this sort of study we want to say that the effect persisted in spite of our best efforts to adjust it away	Thank-you for this comment.<br><br>In small sample sizes with high-dimensionality data, Greenland et al warn that:<br>*"controlling for too many potential confounders can lead to or aggravate problems of data sparsity, or multicollinearity and also brings the risk of misrepresenting associations"* (Greenland, Daniel and Pearce, 2016).<br><br>While Babyak, 2004, advise against data-driven confounder selection when building multivariate models, it must be remembered that we are only presenting bivariate analyses in this paper.<br><br>Additionally, Greenland et al also offers some nuance to advice on pragmatic confounder inclusion:<br>*« …it can be argued that any data-based model reduction will produce biased estimates because it depends on the assumption that it is not necessary to control the omitted*

rather than being able to say that it is not necessary to measure some of the confounders. If the authors feel it is necessary then state why and also state what method was employed to select the variables: forward selection, backward elimination, best ossible subset,	*variables (conditional on control of the included variables). We regard this criticism as somewhat misguided insofar as every epidemiological estimate suffers from some degree of bias from uncontrolled confounders, differential subject selection and measurement error (in both exposures and confounders); the key question is then whether the bias from omitting a variable is of contextual importance. »*<br>(Greenland, Daniel and Pearce, 2016)<br><br>In this high-dimensionality dataset, we feel that contextual importance is key to avoiding "spurious confounding", where for example, it does not make sense and would be an error to control for abdominal pain when assessing vomiting. Nor would it make sense to adjust for CRP when assessing severe illness, as CRP is considered when making this diagnosis.<br><br>As the reviewer knows, confounders follow the same laws of causality as the variables investigated for association that were established by Bradford Hill in 1965 (Bradford Hill, 1965).<br>Firstly, they must they have meaningful independent associations with the outcome and the exposure variables. This independent association with the outcome is screened for in table 3. Features with independent associations are marked here those with contextual and logical associations are controlled (i.e. Age and Seasonality)<br><br>Our dataset is particular, in that the variables were collected using systemic pathways in an electronic decision algorithm, the logic of their relationships with one another can be followed in the protocol of the prospective trial here:<br>https://journals.plos.org/plosmedicine/article/file?id=10.1371/journal.pmed.1002411.s010&type=supplementary<br><br>We feel that we have made appropriate and pragmatic adjustments that deliver a level of complexity that is easily understandable to the reader and robust to the clinical logic of the collected data.
No evidence of effect versus evidence of no effect I felt that in a number of places the authors do not place enough stress on the fact that with 76 children in the LD group some of the confidence intervals are quite wide and we need to be more cautious about sliding from saying there is no evidence of difference to saying there is no difference.	Indeed, we struggled with this sentence, and hope that the adjusted language better portrays this shared view.<br><br>*During a 28-day follow-up period, we did not find evidence of a difference in negative outcomes between febrile children with untreated LD Pf parasitaemia and those without Pf parasitaemia.*

Reviewer 2
We thank the reviewer for their positive appraisal of the article and appreciate the suggestions for improving the references, indeed that are perfectly pertinent.

Comment	Response
Reviewer #2: This is a well performed and well analysed study and a well written article. It adresses a clinically quite important subject/research question, namely the potential benefit vs even negative impact of using the recently developed ultrasensitive mRDTs vs the standard mRDTs - the article provides a rather credible, clear and important answer to this primary research question<br>The article also discusses a few general issues related to parasite densities of Pf infections such as maternal antibodies, sickle cell trait and concomitant infections - which I find adds to my interest in the article	☺
I believe in Title of Table 1 = "sub-cohort" should be ""sub-cohorts"	Thank-you for spotting this.<br>Corrected.
maybe add one ref in rel to the text on withholding malaria treatment to mRDT neg febrile children in malaria endemic countries = Msellem et al PLoS Med 6:e 1000070 , 2009	Agreed and added
definitely consider adding one ref on the effect of viral infections reducing malaria parasite densities in febrile Children = Rooth and Björkman Am J Trop Med 47: 675 , 1992 - this supports the findings in the present article	Agreed and added<br>Thank you!

Reviewer 3

We thank the reviewer for their positive appraisal of our work and we appreciate the comments which we feel improve the clarity of our findings.

<table>
<tr><th>Comment</th><th>Response</th></tr>
<tr><td>Line 11. Capital letters are omitted from the institutional unit.</td><td>Thank-you. Corrected</td></tr>
<tr><td>Statements with respect to "severe illness" and anaemia as "moderate to severe" (as in Table 4A and 4B) are confusing and differ from those in the PLoS Med 2017 paper. Hb concentration values in the manuscript are given as mmol/L whereas values in the 2017 paper are presented as g/L. Secondly, the PLoS Med paper (Fig. 2) defines severe anemia as Hb <60 g/L (Hb 6 g/dL or 3.72 mmol/L). The high density parasitemia patients in this manuscript had a median value of 8.9 mmol/L (14.3 g/dL) and referred to as "severe anaemia". Please clarify what is meant by severe anaemia and what cut off values are used in this urban population. Attention to this detail is particularly important to the Discussion (e.g., lines 381-389).</td><td>Thank-you for spotting this!
Surprisingly, the definitions of severity are not anywhere in the manuscript. They must have been accidently edited out along the way…
We have taken the opportunity to summarise this information clearly in a supplementary table, so that the reader doesn’t have to refer to information in various tables of Keitel et al, 2017.

Table S1. Definition of clinical failure (primary outcome measure). Adapted from Keitel et al. [13]
<table>
<tr><td>**Clinical failure**</td><td>**At any time after initial assessment**</td><td colspan="2">Persistence of new onset of “severe illness” [1]
• Coma
• >2 convulsions within 24h
• Inability to drink or breastfeed
• Tachypnoea [2]
• Tachycardia [3]
• Hypoxemia [4]
Clinical pneumonia [5]
Severe dehydration [8]</td></tr>
<tr><td></td><td>**At day 7**</td><td colspan="2">Fever or temperature > 38°C
Diarrhoea
Serious skin infection [6]
A new significant symptom or sign related to the acute episode but not present at day 0</td></tr>
<tr><td>**Anaemia**</td><td>**Moderate-to-severe**</td><td colspan="2">Haemoglobin >9 g/dl</td></tr>
<tr><td></td><td>**Severe**</td><td>Haemoglobin >6 g/dl</td><td rowspan="5">[1] **Severe illness** any of the conditions highlighted in orange</td></tr>
<tr><td>**Severe malnutrition**</td><td colspan="2">Weight-for-Age Z-score < -3 and/or MUAC <11-5 cm.</td></tr>
<tr><td>[8] **Severe dehydration**</td><td colspan="2">Requiring facility-based treatment
• Not tolerating oral liquids
• Tachycardia</td></tr>
<tr><td>**Severe pneumonia**</td><td colspan="2">Clinical pneumonia [5] AND (Severe respiratory distress [7] OR severe tachypnoea [2] OR Hypoxemia [4])</td></tr>
<tr><td>**Suspicion of meningitis**</td><td colspan="2">Severe lethargy OR neck stiffness OR convulsions</td></tr>
<tr><td>[2] **Tachypnoea**</td><td colspan="3">Respiration rate > 97th percentile for age and temperature</td></tr>
<tr><td>[3] **Tachycardia**</td><td colspan="3">Heart rate > 90th percentile for age and temperature</td></tr>
<tr><td>[4] **Hypoxemia**</td><td colspan="3">Oxygen saturation (SaO2 < 90%)</td></tr>
<tr><td>[5] **Clinical pneumonia**</td><td colspan="3">Either (cough AND tachypnoea [2]) OR (cough AND chest indrawing)</td></tr>
<tr><td>[6] **Serious skin infection**</td><td colspan="3">Requiring antibiotic based treatment</td></tr>
<tr><td>[7] **Severe respiratory distress**</td><td colspan="3">Speaks only single words OR grunts OR speaks short phrases only OR short cries AND chest indrawing</td></tr>
</table>

Here we highlight the conditions included in the definition of “severe illness” (orange) and clearly differentiate severe and moderate-to-severe anaemia.

As shown in the table, the definition for severe anaemia is unchanged from Keitel et al, 2017 (<60 g/L or <6 g/dL).
The “moderate to severe” anaemia category is now clearly defined (<90 g/L or <9 g/dL) and also report units in g/dl.</td></tr>
<tr><td>Severe malnutrition is a major confounder in the analysis. More detail on the criteria for the diagnosis of "severe malnutrition" in the ePOCT algorithm is needed.</td><td>Agreed.
This is definition is now included in the supplementary table depicted above.
FYI the definition is: Weight-for-Age Z-score < -3 and/or MUAC <11.5 cm</td></tr>
<tr><td>Acronyms are over used. RDT and PCR are generally understood terms but readability would be greatly improved by avoiding terms such LD, ND, us-qPCR+ vs. qPCR- etc. Terminology in the manuscript with respect to "high density", "low density" parasiteimia, etc. is confusing and potentially misleading to some readers. This is particularly true in settings in sub-Saharan Africa where the Pf biomass is readily detectable by microscopic inspection of blood smears and RDTs are not routinely performed in order to reduce cost.</td><td>Agreed, we have reviewed the text in detail to limit the use of us-qPCR, sd-mRDT, us-mRDT to the figures/tables only.
In the text these are now referred to in full i.e.
Ultra-sensitive qPCR, standard mRDT and ultra-sensitive mRDT.
Thus, now the only acronyms that are repeatedly used are those describing the main cohorts (ND, LD, HD for non-detectable, low-density and high-density)
By using low and high density we are following the terminology of previous publications.</td></tr>
<tr><td>Table 1 is unnecessary and could be omitted</td><td>As the ND, LD and HD groups are being kept, we feel it is critical to keep table 1 to best clarify their composition and reduce confusion.</td></tr>
</table>

Reviewer 4

We thank the reviewer for their thorough review of the manuscript and are happy to have been able to address the issues raised.

The paper is written in a rather unconventional way. Nothing wrong with innovation but I have to admit I found it hard to find my way around this paper. More confusion comes from near complete similarity of standard RDT+ and us-RDT+ study participants. Since the difference in sensitivity between the two RDTs is minimal, despite the name "ultrasensitive" there is very little gained from testing with us-RDTs. The us-RDTs missed the majority of the low density infections detected by PCR. Hence the inclusion of a patient group "us-RDT+" adds no information but adds considerable confusion. Would the authors consider omitting the data on us-RDTs since it contributes nothing meaningful to description of clinical sequelae of low density infections? I understand the desire to publish the data but the inclusion of the usRDT data do not make the paper stronger.	We definitely agree with the reviewer.<br>Indeed, all the ultra-sensitive mRDT data have been removed from further analyses and results only appear in the the supplementary table S2 for interested readers.<br><br>The reviewer identifies a noteworthy irony that the ultra-sensitive mRDT performs no better than the standard test.<br>We feel that this is extremely important information to report as the tests are none the less sold as "ultra-sensitive" and thus the information that they may not provide any further sensitivity in such an epidemiological setting is of high clinical interest.
Provide total numbers included in each of the two groups: sd-mRDT-/us-qPCR+, sd-mRDT-/us-qPCR-, sd-mRDT+/us-qPCR+.	This was previously omitted due to word limitations.<br>Added back now
Abstract: "Pf positivity rate was 7.1% and 9.8% by sd-mRDT and us-qPCR respectively." What % of usRDTs were positive?	According to the reveiwers request above to limit the importance of us-mRDT, we will refrain from reporting it in the abstract but rather only mention the results in the relevant section in the results and discussion.<br><br>***Results (Pf parasitaemia prevalence)***<br>*Pf positivity was 7.1% (n=199/2801) by standard mRDT, 7.5% (n=209/2801) by ultra-sensitive mRDT and 9.8% (n=274/2801) by ultra-sensitive qPCR. Standard mRDT and ultra-sensitive qPCR results are presented in **Table 2**, ultra-sensitive mRDT results can be found in the supplement, **Table S2**.*
Intro<br>"A new easy-to-use and affordable ultra-sensitive mRDT (us-mRDT) was developed (Alere™, Abbott Diagnostics) able to detect the Pf Histidine-Rich Protein-2 (HRP-2) with ten-fold greater sensitivity to that of sd-mRDTs." I do not understand why the authors use reference #8 if their own group has made much more realistic estimates of the added sensitivity of us-RDT? "us-RDT identified few additional P. falciparum-positive patients as compared to co-RDT (276 vs 265 parasite-positive patients detected), with only a marginally greater sensitivity (75% vs 73%), using us-qPCR as the gold standard …" (Hofman et al JID 2019).	Indeed.<br>This reference was actually just above. However it is certainly relevant to mention this here and we have now added it as suggested.<br><br>*Our group, however has reported only marginal improvements on sensitivity using ultra-senstivie qPCR as a gold standard (75% vs 73%) [6].*
"As many of the LD infections are also asymptomatic, these highly sensitive tests resurrect Koch's postulates of what defines a "pathogen", and at what concentration should a detected parasite be considered "disease-causing", necessitating the implementation of preventive or therapeutic interventions."<br>1) resurrect Koch's postulates - if they have not died they do not need resurrection.<br>2) parasitaemia is hardly physiologic - if possible the patient with parasitaemia should be treated - even if interventions do not benefit the patient	1) Alright. The word resurrect has been changed to "challenge" to be a bit more traditional<br>2) That is just the question that this manuscript aims to raise. Should they be treated? We would still require further studies on the capacity of community transmission of this trace-carriage, but our paper indicates that this treatment might not have a clinical motivation for the patient.

<table>
<tr><td>directly, the patient should be treated to prevent transmission?</td><td></td></tr>
<tr><td>"It thus becomes important to assess at which point this gain in sensitivity surpasses its clinical benefit, …" Ultrasensitive diagnostics have not been developed for clinical management of patients. Please clarify this point.</td><td>Detecting “incidental Pf parasitaemia in a febrile child may distract clinicians from diagnosing other serious and treatable co-infections such as bacterial sepsis or meningitis“

This sentence is placed just afterwards for clarification.</td></tr>
<tr><td>The authors write at the end of the intro: "Thus these analyses aim to fill the gaps highlighted by the WHO technical committee for policy recommendations on highly sensitive point-of-care Pf malaria diagnostics by elucidating the prevalence and clinical presentation of LD parasitaemia detectable only by ultra-sensitive tools such as us-qPCR and us-mRDT and measuring its impact on clinical outcomes in febrile outpatients." Would it be possible to state clearly what the research question was?</td><td>The research question is: what is potential clinical impact of lowering the parasitaemic threshold in future diagnostics ?

This is stated just above the paragraph in question</td></tr>
<tr><td>Results:
Figure 1: please clearly indicate in the chart how many study participants were tested with 1) standard RDT, 2) us-RDT, 3) us-PCR?</td><td>Please refer to Table 2 and Table S2</td></tr>
<tr><td>Please add a table describing the demographic characteristics of 3 patient groups: low density, high density and no infections. In the table please indicate the numbers testing positive with which test (sd-RDT, us-RDT, usPCR.</td><td>Please refer to Table 3.
As per the reviewer’s recommendation, we do not include us-mRDT data in the main results.</td></tr>
<tr><td>If the authors insist to include the us-RDT data in the paper Table 2 is inappropriate and should be replaced with table S2.</td><td>As per the reviewer’s suggestion, the us-RDT data is limited to the text and only one reference to the Table S2 is provided for readers requiring further clarification.</td></tr>
<tr><td>How was "severe illness defined"?</td><td>We have taken the opportunity to summarise the clinical definitions more clearly in a supplementary table, so that the reader doesn’t have to refer to information in various tables of Keitel et al, 2017.

Table S1. Definition of clinical failure (primary outcome measure). Adapted from Keitel et al. [13]
<table>
<tr><td rowspan="2">Clinical failure</td><td>At any time after initial assessment</td><td colspan="2">Persistence of new onset of “severe illness” [1]
• Coma
• >2 convulsions within 24h
• Inability to drink or breastfeed
• Tachypnoea [2]
• Tachycardia [3]
• Hypoxemia [4]
Clinical pneumonia [5]
Severe dehydration [8]</td></tr>
<tr><td>At day 7</td><td colspan="2">Fever or temperature > 38°C
Diarrhoea
Serious skin infection [6]
A new significant symptom or sign related to the acute episode but not present at day 0</td></tr>
<tr><td rowspan="2">Anaemia</td><td>Moderate-to-severe</td><td colspan="2">Haemoglobin >9 g/dl</td></tr>
<tr><td>Severe</td><td>Haemoglobin >6 g/dl</td><td rowspan="5">[1] Severe illness
any of the conditions highlighted in orange</td></tr>
<tr><td>Severe malnutrition</td><td colspan="2">Weight-for-Age Z-score < -3 and/or MUAC <11-5 cm.</td></tr>
<tr><td>[8] Severe dehydration</td><td colspan="2">Requiring facility-based treatment
• Not tolerating oral liquids
• Tachycardia</td></tr>
<tr><td>Severe pneumonia</td><td colspan="2">Clinical pneumonia [5] AND (Severe respiratory distress [7] OR severe tachypnoea [2] OR Hypoxemia [4])</td></tr>
<tr><td>Suspicion of meningitis</td><td colspan="2">Severe lethargy OR neck stiffness OR convulsions</td></tr>
<tr><td>[2] Tachypnoea</td><td colspan="3">Respiration rate > 97th percentile for age and temperature</td></tr>
<tr><td>[3] Tachycardia</td><td colspan="3">Heart rate > 90th percentile for age and temperature</td></tr>
<tr><td>[4] Hypoxemia</td><td colspan="3">Oxygen saturation (SaO2 < 90%)</td></tr>
<tr><td>[5] Clinical pneumonia</td><td colspan="3">Either (cough AND tachypnoea [2]) OR (cough AND chest indrawing)</td></tr>
<tr><td>[6] Serious skin infection</td><td colspan="3">Requiring antibiotic based treatment</td></tr>
<tr><td>[7] Severe respiratory distress</td><td colspan="3">Speaks only single words OR grunts OR speaks short phrases only OR short cries AND chest indrawing</td></tr>
</table>

Here we specifically highlight the conditions included in the definition of “severe illness” (orange).</td></tr>
<tr><td>Discussion
"LD infection prevalence fell to zero during the non-malaria season, perhaps an indication that LD infection was not functioning as an off-season reservoir, …" considering the limited</td><td>Certainly agreed.
We specify : “albeit that larger epidemiological surveys are required before such conclusions can be made” directly after this statement for clarification.</td></tr>
</table>

sample size of the surveys the conclusions may be premature. It would take much more complete surveys of the entire population to justify the conclusion that low density infections do not contribute to transmission.	
"Lower endemicity settings also have a lower diversity of parasites to which inhabitants may be more readily immune, and thus also partly explain the higher LD prevalence in recent low-endemicity settings." Shouldn't immunity clear infections and reduce the parasite prevalence?	Thank-you for this pertinent question.<br>Immunity is not binary. The grey zones between “immune” and “vulnerable” allow pathologic carriage and is also responsible for tolerating the symbiosis of our gut microbiota.<br>The equilibrium stuck within each patient is unique, but it is accepted that low-level immunity may be responsible for LD plasmodia carriage as cited below this text in the manuscript
"This study shows that, at least in a moderate malaria endemicity setting, low-density (LD) Pf parasitaemia infections undetected by mRDT did not present acute health concerns in children in an outpatient setting." Please consider rephrasing? The way the sentence currently reads the reader could think that prior to this study low density infections required acute health care. This is misleading. It would be correct to state that the study confirmed that low density infections do not require acute interventions but when detected should be treated if only to prevent transmission.	This study provides no evidence on the transmissibility of these trace “infections” (which may not even represent live parasites but rather just their genetic remnants incidentally detectable in recently infected patients with no capacity of transmission).<br><br>Thus, we strictly cannot recommend the use of treatment in these patients especially in the moderate endemicity setting of Tanzania which is not currently following a “malaria elimination” strategy.<br><br>The WHO defines a malaria case according to their aims of local control. The case definition is more sensitive in areas where “elimination” is the goal (as opposed to “control”). How ultrasensitive diagnostics will be used in the definition of a “treatable case” requires large scale epidemiologic surveys.<br><br>We address this issue in the discussion.
